# Nanocomposite Coatings Based on Polyvinyl Alcohol and Montmorillonite for High-Barrier Food Packaging

**DOI:** 10.3389/fnut.2022.790157

**Published:** 2022-03-07

**Authors:** Stefan Schiessl, Esra Kucukpinar, Stéphane Cros, Oliver Miesbauer, Horst-Christian Langowski, Peter Eisner

**Affiliations:** ^1^TUM School of Life Sciences, Technical University of Munich, Freising, Germany; ^2^Fraunhofer Gesellschaft FhG, Fraunhofer Institute for Process Engineering and Packaging IVV, Freising, Germany; ^3^Département Des Technologies Solaires, Université Crenoble Alpes, Commissariat àl'énergieatomique et aux énergies alternatives, Le Bourget-du-Lac, France; ^4^Steinbeis-Hochschule, Faculty of Technology and Engineering, Dresden, Germany

**Keywords:** barrier coating, montmorillonite, halloysite, tortuous path, polyvinyl alcohol, nanocomposite, helium permeability, oxygen permeability

## Abstract

Materials with high barrier properties against oxygen are required for the packaging of many sensitive foods. Since commodity polymers lack these properties, additional barrier materials are used in plastic-based barrier packaging. These are usually more expensive than commodity polymers and, in higher fractions, also make recycling more difficult. Current developments, therefore, aim at barrier layers that are as thin as possible but retain the barrier properties. One approach is to incorporate nanoparticles into these layers. In this study, the barrier properties of nanocomposite coatings, consisting of unmodified polyvinyl alcohol (PVA), and dispersed stick-shaped halloysite (Hal) or platelet-shaped montmorillonite (MMT) silicate nanoparticles, were investigated. The PVA was dissolved in aqueous nanoparticle dispersions, which were prepared by mechanical shearing, to produce the so-called “nanolacquer.” Nanolacquers with nanoparticle concentrations of 7, 30, and 47 vol% with respect to PVA were applied in a single process step with *k*-bar on a polypropylene substrate film. The integration of 30 vol% platelet-shaped MMT enhances the barrier performance in comparison to pure PVA by a factor of 12 and 17 for oxygen and helium, respectively. Scanning electron microscopy (SEM) shows a homogeneous distribution and a parallel alignment of the nanoparticles within the coated layer. An increase in the crystallinity of PVA was observed due to the nanoparticle integration as demonstrated by *x*-ray diffraction (XRD) measurements. The investigation by Fourier transform infrared (FTIR) spectroscopy and the activation energy of the permeation coefficient indicate an interaction between the nanoparticles and the PVA. The theoretically calculated values for barrier enhancement accord well with the experimental values, which emphasizes that the gas barrier improvement for oxygen and helium is mainly dominated by the tortuous path effect.

## 1. Introduction

Some sensitive foods are described by consumers as sensory impaired already at an oxygen intake of less than 1 $\frac{\text{mg}}{\text{kg}}$ (1). For such products, packaging materials with oxygen permeability down to 0.1 $\frac{{\text{cm}}^{3}\;\left(\text{STP}\right)}{{\text{m}}^{2}\;\text{d}\;\text{bar}}$ are needed to ensure a sufficiently long shelf life (2). If one wants to achieve high barrier properties, lightweight, and good recyclability for the corresponding packaging at the same time, different solutions are available. The most common are polymer barrier layers made of polyvinyl alcohol (PVA).

These PVA barrier layers are targeted to be as thin as possible without having to sacrifice the barrier properties, because of two main reasons. First, the cost of PVA can be about three to five times higher than for polyolefins that are typically used in flexible packaging applications. Second, the PVA interferes with the recycling of polyolefins. Since the incorporation of platelet-shaped nanoparticles has already shown a significant improvement in many polymers, especially in the oxygen barrier properties (3), this approach was also pursued in this study.

Polyvinyl alcohol provides one of the lowest gas permeability among polymers (4). Its excellent oxygen (O_2_) barrier property is - in addition to its flexibility, transparency, toughness, non-toxicity, water solubility, and chemical resistance—the reason why it is a standard polymer for high-barrier food packaging (5, 6). Due to the sensitivity of its oxygen barrier performance toward the high humidity levels, PVA layers are commonly used behind a polypropylene or a polyethylene layer in typical multilayered flexible packaging applications.

The measured oxygen transmission rate is highly dependent on the degree of hydrolysis, the molecular weight, the curing, the measuring conditions, and above all the composition. PVA compositions with an ethylene content of less than 10 %, which are still water soluble, are called PVA in the literature, instead of the more accurate abbreviation EVOH, for ethylene vinyl alcohol co-polymer. The reason, therefore, is that nearly all PVA coatings contain at least a small amount of ethylene, to reduce the melting point of the PVA and increase the temperature gap between the melting point and the decomposition temperature for easier processing of the PVA (7, 8).

Hence, the oxygen permeability coefficients for PVA reported in the literature vary significantly. A summary of several approaches and the corresponding values is given in Table 1. While one of the lowest values, 0.3 $\frac{{\text{cm}}^{3}\;\left(\text{STP}\right)\;1\;\mu \text{m}}{{\text{m}}^{2}\text{d}\;\text{bar}}$, was obtained by Tsurko et al. (9) with 100 to 1,000 times spray coating of PVA on PET, leading to a dry film thickness of 700 nm, values of higher than 100 $\frac{{\text{cm}}^{3}\;\left(\text{STP}\right)\;1\;\mu \text{m}}{{\text{m}}^{2}\text{d}\;\text{bar}}$ are reported for free-standing films (10–13). But apart from these, a typical value for one time coating on flexible films, which was reported as 1 to 6 $\frac{{\text{cm}}^{3}\;\left(\text{STP}\right)\;1\;\mu \text{m}}{{\text{m}}^{2}\text{d}\;\text{bar}}$, has been set as a comparative value within this study (14–18).

**Table 1:** Overview of oxygen permeability values *P*_O_2__ for PVA and PVA in combination with silicate nanoparticles described in Section 1.

	***P*_O_2__**	**Approach**	**Source**
	$\frac{{\text{cm}}^{3}\;\left(\text{STP}\right)\;1\;\mu \text{m}}{{\text{m}}^{2}\text{d}\;\text{bar}}$		
PVA	0.300	700 nm dry coating layer thickness on PET substrate film, coated with spray coating (100 to 1000 times)	(9)
	1.00	6 μm dry coating layer thickness on PET substrate film, coated with k-bar	(14)
	1.20	4.4 μm dry coating layer thickness on PET substrate film, coated with k-bar	(15)
	4.30	2 μm dry coating layer thickness on PET substrate film, coated with k-bar	(18)
	135	free standing film with 30 μm dry thickness poured in Petri dish and de-formed	(12)
	238	free standing film with 140 μm dry thickness purred in Petri dish and de-formed	(13)
	358	free standing film with 60 μm dry thickness coated with k-bar on glass and peeled off	(11)
PVA in combination with nanoparticles	0.001	synthesized silicate particles with high aspect ratio mixed with PVA and spray coated (100 to 1000 times) on PET	(9)
	0.002	slurry of PVA and MMT poured onto PET sitting in a Petri dish	(26)
	0.010	mixture of MMT, Laponite, and PVA coated 5 times *via* dip coating of PET	(25)
	0.025	modification of PVA with vinyl acetate and iactonic acid and mixing with MMT, coated with k-bar on PET	(14)
	0.200	30 layers in summary, alternating PVA and MMT dispersion coated with k-bar on PET	(23)
	0.800	12 layers in summary, alternating PVA and MMT dispersion coated *via* ink-jetting on PET	(24)

By the integration of nanoparticles, the abovementioned properties have been enhanced. Coatings with nanocomposite materials provide, e.g., enhancements in antimicrobial, self-healing, flame retardant and barrier properties (19, 20). The oxygen barrier improvement for PVA as a polymer matrix, due to the addition of platelet-shaped silicates, has already been widely studied (see Table 1).

One of the approaches is the development and synthesis of silicate particles, which are nearly impermeable for molecular species, with the highest possible aspect ratio. The aspect ratio is the ratio of the longest and the shortest dimension of a particle. The higher this ratio is, the better is the barrier that can be achieved (21, 22) synthesized, e.g., silicate nanoparticles with an aspect ratio of up to 20,000 (length of 20 μm and width of 1 nm). Nanocomposites with these particles provided one of the lowest published permeability coefficients with 0.001 $\frac{{\text{cm}}^{3}\;\left(\text{STP}\right)\;1\;\mu \text{m}}{{\text{m}}^{2}\text{d}\;\text{bar}}$ at 23°C and 50 % RH for a 100 to 1,000 times spray coating on PET by Tsurko et al. (9).

Another approach is a layer-by-layer application of PVA and montmorillonite (MMT) layers. Ben Dhieb et al. (23) reached an oxygen permeability coefficient of 0.2 $\frac{{\text{cm}}^{3}\;\left(\text{STP}\right)\;1\;\mu \text{m}}{{\text{m}}^{2}\text{d}\;\text{bar}}$ at 25°C and 0 % RH by an alternating structure of 15 PVA and 15 MMT layers, using a doctor blade coating process. Dabbaghianamiri et al. (24) used an inkjet printing process to produce 6 bilayers. The final value was 0.8 $\frac{{\text{cm}}^{3}\;\left(\text{STP}\right)\;1\;\mu \text{m}}{{\text{m}}^{2}\text{d}\;\text{bar}}$ at 23°C and 40 % RH.

One practical approach, for the preparation of PVA-based nanocomposite coatings, focuses on using commercially available clay particles and PVA lacquers, and only a single-layer coating on the respective substrate. For mixtures of PVA with increasing ratios of kaolin (phyllosilicate like MMT with a slightly different structure), Nyflätt et al. (12) did not observe any improvement on the barrier properties of the coatings on PET at a thickness of 1.8 μm. Meng et al. (25) did not find a barrier improvement due to MMT integration for one-time dip-coated PET films. The films have been dipped in PVA and a nanocomposite lacquer of PVA and MMT at a mixing ratio of 1:2. The dry coating layer thickness was 0.8 μm.

In these studies using commercially available clay particles and PVA, some further investigations were still required; for example, Meng et al. (25) mixed MMT with laponite and PVA in equal parts and increased the number of layers to five, leading to a total dry coating layer thickness of 4.0 μm and an oxygen permeability coefficient of 0.01 $\frac{{\text{cm}}^{3}\;\left(\text{STP}\right)\;1\;\mu \text{m}}{{\text{m}}^{2}\text{d}\;\text{bar}}$ at 23°C and 0 % RH. Grunlan et al. (14) mixed PVA with vinyl acetate and iactonic acid to get a terpolymer with an intrinsically high oxygen permeability coefficient of 0.5 $\frac{{\text{cm}}^{3}\;\left(\text{STP}\right)\;1\;\mu \text{m}}{{\text{m}}^{2}\text{d}\;\text{bar}}$ at 23°C and 55 % RH. This value could be decreased by a factor of 20 by mixing with 20 wt% MMT particles. A high oxygen permeability coefficient of 0.002 $\frac{{\text{cm}}^{3}\;\left(\text{STP}\right)\;1\;\mu \text{m}}{{\text{m}}^{2}\text{d}\;\text{bar}}$ at 23°C and 0 % RH was reached by Song et al. (26) with a quite simple process: They poured a slurry of PVA and 50 % MMT onto a 179 μm thick PET substrate and let it dry at room temperature for 6 h.

Although the barrier improvement of PVA coatings with silicate nanoparticles has been proven and very high oxygen barrier performance has already been reported in the literature, the usage of PVA silicate nanocomposites in the packaging industry is still rare. The reasons for this are manifold: either the synthesis of silicate particles is extensive, the number of coating layers is high, the curing time is not adjusted to industrial scale, the substrate material is not the material of interest for the food packaging industry, the barrier properties can only be achieved at low humidity, or there are currently no available green and effective exfoliation methods for the particles. Besides the economic reasons, further scientific knowledge is needed. Factors such as crystallinity, tortuosity, interactions between particles and the polymer need to be further investigated and identified (20, 27).

The aim of this study is a deeper understanding of gas permeation mechanisms in silicate nanocomposites. Therefore, several factors have been taken into account. First of all, two different permeants with different gas kinetic diameters, helium (2.6 Å) and oxygen (3.5 Å), were used (28). The bigger oxygen molecules permeate predominantly through macro-defects (> 1 nm), whereas the permeation of helium is mostly determined by the permeation through nano-defects (< 1 nm) (29–31).

To gain a better understanding of the tortuosity effect, two differently shaped silicate nanoparticles were selected: a stick-shaped Halloysite (Hal), and a platelet-shaped MMT. MMT is a 2:1 type, meaning two SiO-tetrahedra layers flank a central AlO-octahedra layer. Hal is a 1:1 type, like kaolin, but rolled to a nanotube with the SiO-tetrahedra layer outside and the AlO-octahedra layer inside (32).

Since the exfoliation of clay particles to nanoparticles is the crucial step during the production of a nanolacquer, because only well-exfoliated and well-dispersed nanoparticles can improve the barrier properties (20), two different mechanical dispersing techniques were used and the results were compared: a mechanical stirrer and a planetary ball mill. Compared to the typically used mechanical stirrers, the usage of ball mills in the field of nanocomposites is only starting (33). The colliding surfaces in a ball mill subject the particles to mechanical load. This mechanical load, leading to the fracturing and exfoliation, appears very homogeneously in the whole dispersion during the milling process. A mechanical stirrer, in comparison, has one rotating head that applies the shear force to the liquid, with only random direct contact with the particles (34). The resulting dispersions from the dispersing instrument and the planetary ball mill were used as the basis for the nanolacquer and the effect of the mechanical dispersing technique on agglomerations and gas barrier properties is compared.

Finally, the barrier improvement for the respective nanoparticle shape and concentration was calculated based on geometrical factors only and compared with the measured permeability values to identify whether the tortuosity or the structural changes within the PVA resin is the dominating factor for the barrier improvement.

## 2. Materials and Methods

### 2.1. Materials

Two different types of naturally abundant clay-based silicate types were used to exfoliate nanoparticles, MMT and Hal. The MMT was purchased as CLOISITE Na^+^ from BYK Additives and Instruments, Wesel, Germany. Dry ball-milling of this MMT was performed without the addition of any solvent by a proprietary process of MBN Nanomaterialia, Carbonera TV, Italy (35). The powder obtained was then agglomerated, in form of granules, by using a small amount of water, highly reducing the dustiness of the product and, thus, providing operational safety. The Hal was purchased as Hal Clay MF7 from DURTEC, Neubrandenburg, Germany. These two powder types were then mixed with water according to the procedures as described in Section 2.1.1 to obtain the feed-stock dispersion for the coating processes.

PVA, with the trade name Exceval AQ 4104, from Kuraray (Frankfurt, Germany), was the polymeric basis for the lacquers. Since the ethylene content of this material is below 10 %, it is called polyvinyl alcohol in the technical data sheet, although its real chemical composition is an atatic copolymer of ethylene and vinyl alcohol. The molecular weight of AQ 4104 is 27.000 $\frac{\text{g}}{\text{mol}}$ and the degree of hydrolysis is 98 to 99 mol% (36). The glass transition temperature, *T*_g_, is 74.8°C, the crystallization temperature is 193.3°C, and the melting temperature is 220°C (37).

Biaxially oriented Polypropylene (BoPP) sheets (30 μm in thickness) were purchased as transparent, non-sealable (referred to as TNS) film from Taghleef Industries (San Giorgio, Italy).

To assure a suitable and repeatable wetting, the substrate was pretreated with a modified polyethylenimine (PEI) primer, ORGATIX WS-680A from Matsumoto Fine Chemical Co. (Chiba, Japan). The modification is a crosslinking with a water soluble titanate compound. The primer provides a solid content of 9 % in weight (38).

#### 2.1.1. Preparation of Nanoparticle Dispersion

To exfoliate the clay particles to nanoparticle size, two devices, both providing high mechanical shearing, were used; a high-performance mechanical stirrer (T 25B ULTRA-TURRAX^®^, IKA Labortechnik, Staufen, Germany) and a planetary ball mill (PULVERISETTE 6, FRITSCH, Idar-Oberstein, Germany). Four different types of aqueous nanoparticle dispersions, each at a solid content of 5 wt%, were produced using either the MMT or Hal powders, each by using one of the two dispersing processes. These four dispersion types are defined as MMT dispersion-UT, MMT dispersion-BM, Hal dispersion-UT, and Hal dispersion-BM. UT and BM are the abbreviations used for ULTRA-TURRAX^®^ and planetary ball mill processes, respectively. The preliminary trials of the authors have shown that, as the nanoparticle concentration increases, the gas barrier performance also increases. On the other hand, if the solid content is increased above 5 wt%, the dispersion becomes pasty. The solid content of 5 wt% was chosen in this study due to the fact that it is the highest possible amount at which the viscosity of the material still allows a homogeneous mixture in PVA as discussed in Section 2.1.4. Based on this, 190 ml of water and 10 g of nanoparticles were mixed for 1 min at 1.000 rpm in a laboratory glass bottle with the ULTRA-TURRAX^®^ (UT). For the exfoliation with the planetary ball mill (BM), 190 ml of water and 10 g of nanoparticles were put in a zirconium oxide cup together with 500 g of zirconium oxide balls (each ball 3 mm in diameter) and rotated at 400 rpm for 1 h. Several exfoliation times for UT and BM have been tested for both processes and further increase of this parameter did not lead to different results, as well as a change of rotation time (UT and BM) and ball size (only BM), therefore, this is not shown in this manuscript.

#### 2.1.2. Fabrication of Nanolacquer

To produce the so-called “nanolacquer,” 4 g of PVA granulate were added to the nanoparticle dispersions and slightly diluted with water to obtain the nanoparticle to PVA mixing ratios of 1:6, 1:1, and 2:1 in weight (refer to Table 2). This represents the minimum, medium, and high volume fractions of 7, 30, and 47% for the silicate particles calculated with the densities of 2.86 $\frac{\text{g}}{{\text{cm}}^{3}}$ and 1.22 $\frac{\text{g}}{{\text{cm}}^{3}}$ for MMT and for pure PVA, respectively (36, 39). To start the dissolving process of PVA, the mixture was stirred in a closed beaker with a magnetic stirrer for 30 min at 400 rpm. Subsequently, the mixing rate was reduced to 150 rpm and heated up to 90°C for 120 min until the PVA was dissolved completely. This lacquer was then cooled down to room temperature under constant shearing at 150 rpm and degassed in a pressure tank for about 2 h.

**Table 2:** Nanolacquer formulations using two types of exfoliated nanoparticles (NP) of two different processes, in PVA polymer, each at three different NP:PVA ratios in weight.

**Sample code**	**Mixing ratio**	**NP weight**	PVA weight^*^	**Total solid content**
	**(NP : PVA)**	**(g)**	**(g)**	**(wt%)**
PVA-ref	0:1	-	8.0	8.0
MMT-UT-30	1:1	4.0	4.0	8.0
MMT-UT-7	1:6	0.7	4.0	4.7
MMT-UT-47	2:1	8.0	4.0	7.3
MMT-BM-30	1:1	4.0	4.0	8.0
MMT-BM-7	1:6	0.7	4.0	4.7
MMT-BM-47	2:1	8.0	4.0	7.3
Hal-UT-30	1:1	4.0	4.0	8.0
Hal-UT-7	1:6	0.7	4.0	4.7
Hal-UT-47	2:1	8.0	4.0	7.3
Hal-BM-30	1:1	4.0	4.0	8.0
Hal-BM-7	1:6	0.7	4.0	4.7
Hal-BM-47	2:1	8.0	4.0	7.3

* *For these series of tests, the PVA weight in the final nanolacquer was kept constant*.

#### 2.1.3. Pretreatment of Substrate Film

As described in Section 2.1 the PEI primer, ORGATIX WS-680A, was used to pretreat the substrate chemically. An amount of 30 ml primer was mixed with 970 ml of ethanol (>99.7 %, ethanol absolute, VWR CHEMICALS, Darmstadt, Germany), leading to a solid content of 0.3 wt%. The mixture was applied using a reverse gravure coating process (JWS Maschinenfabrik GmbH, Sinsheim, Germany) at the Fraunhofer-IVV at a web-speed of 4 m/min, and dried at 80°C for 50 s. The theoretical dry layer thickness has been calculated based on the density of 0.789 $\frac{\text{g}}{{\text{cm}}^{3}}$, and with the given solid content of 0.3 wt% for this formulation. It has given a dry layer thickness value of 16.6 nm. The surface energy after pretreatment was measured to be more than 44 $\frac{\text{mN}}{\text{m}}$ by means of test inks (Teststifte PINK, arcotest, Mönsheim, Germany, according to DIN ISO 8296) (40). This is sufficiently high for the wettability of PVA-based nanolacquers in comparison to untreated PP with 30 $\frac{\text{mN}}{\text{m}}$. After the roll-to-roll pretreatment with PEI primer, the BoPP film was cut into A4 sized sheets for coatings as described in Section 2.1.4.

#### 2.1.4. Coating of Substrate With Nanolacquer

The pure PVA and the nanolacquer formulations shown in Table 2 were used to coat the chemically pretreated BoPP substrate. The coatings were applied by a lab-coating unit CUF 5 (Sumet Messtechnik, Denklingen, Germany) with an actuation speed of 30 $\frac{\text{mm}}{\text{s}}$ and a contact pressure of 30 N. The coating was performed using the k-bar providing a wet layer thickness of 33 μm. The drying process took place directly after the application of the wet lacquer at 80°C in a built-in convective dryer for 2 min.

#### 2.1.5. Preparation of Nanocomposite Free-Standing Films

For the X-ray diffraction (XRD) measurements, free-standing films of pure PVA and nanocomposites were produced, since the BoPP signal was overlapping with the signals of PVA and silicate particles. Therefore, the aforementioned lacquers were coated on a polytetrafluoroethylene (PTFE) film after corona pretreatment according to the procedure in Section 2.1.4, but with several repetitions until a dry layer thickness of 15 μm is reached for each formulation. After 1 week, the coating layers were peeled from the PTFE. A minimum thickness of 15 μm was necessary since the films cannot be handled below this thickness.

### 2.2. Methods

#### 2.2.1. Asymmetrical Flow Field-Flow Fractionation (AF4)

The particles were separated according to size by means of AF4 measurements with the AF2000 MT Series mid temperature (Postnova Analytics, Landsberg am Lech, Germany). The system is equipped with a 350 μm channel and a cellulose membrane (cut-off: 5 kDa, Postnova Analytics). The channel was constantly maintained at 40°C. Ultrapure water with a conductivity of <0.055 $\frac{\mu \text{S}}{\text{cm}}$ was prepared in-house using a TKA GenPure ultrapure water system (TKA Wasseraufbereitungssysteme GmbH, Niederelbert, Germany) and used as flowing liquid for the AF4. The channel flows were controlled by AF2000 Control Program software (Postnova Analytics). Aqueous MMT dispersions were injected into the channel at a flow of 0.2 ml/min with a focus flow of 1.3 ml/min. During the injection time of 8 min, and an additional transition time of 1 min, the cross-flow was kept constant at 1.0 ml/min. After transition, the cross-flow was eliminated within 25 min using a parabolic flow profile (power gradient of 0.2). The main channel flow was kept constant at 0.5 ml/min during the entire run. Sample amounts of 50 μl were injected with a PN5300 series autosampler (Postnova Analytics).

#### 2.2.2. Multi-Angle Laser Light-Scattering Spectrometry (MALLS)

A 21-angle MALLS detector PN3621 (Postnova Analytics) was used for the determination of MMT particle size, controlled by AF2000 Control Program software (Postnova Analytics) (41). The size of the eluting particles was calculated as the radius of gyration *r*_g_ using a random coil fit. The calculated *r*_g_ was converted into a geometrical radius *r*_geo_ using the equation developed by Andersson et al. (42).


(1) $$\begin{array}{l} r_{\text{g}}^{2}=0.6\cdot r_{\text{geo}}^{2}. \end{array}$$

The particle extension in length *L* and thickness *T*, assuming that the MMT particles are symmetric, is calculated as


(2) $$\begin{array}{l} 2\cdot r_{\text{geo}}=L=T. \end{array}$$

The detector was directly coupled to the AF4 system as discussed in Section 2.2.1 and was operated at λ = 532 nm and 25 mW laser power.

#### 2.2.3. Measurement of Oxygen Permeability

The oxygen permeability, *Q*_total_, for coated substrates was measured according to the DIN 53380-3 standard with the oxygen-specific carrier gas method (43). The measurements were carried out with an OX-TRAN 2/21 OTR Analyzer (AMETEK MOCON, Minneapolis, USA) at 23°C and 50 % RH, and afterward also at 23, 25, 30, 35, and 40°C and 0 % RH. If the respective transmission rate was constant for at least 10 h, the measurement was stopped assuming that the steady state was established. A 2-fold determination for all coatings was carried out. The activation energy of the permeation coefficient *E*_A_ was determined *via* an Arrhenius plot


(3) $$\begin{array}{l} P=P_{0}\cdot e^{-\frac{E_{\text{A}}}{RT}} \end{array}$$

by measuring the permeability coefficient *P* at the aforementioned five temperatures at 0 % RH (44). The oxygen permeability of the deposited nanocomposite layer, *Q*_NC_, was calculated using the following equation


(4) $$\begin{array}{l} \frac{1}{Q_{\text{total}}}=\frac{1}{Q_{\text{Subs}}}+\frac{1}{Q_{\text{NC}}}. \end{array}$$

In Equation (4), *Q*_Subs_ is the oxygen permeability of the BoPP substrate, and *Q*_total_ is the total permeability of the coated BoPP substrate. Since the permeability strongly depends on the layer thickness *d*, the permeability values were converted to the corresponding permeability coefficients *P* according to Equation (5) (45).


(5) $$\begin{array}{l} P=Q\cdot d. \end{array}$$

A common way to compare the different modifications for gas barrier improvement is the calculation of the barrier improvement factor, *BIF*. As shown in Equation (6), the *BIF* is calculated from the ratio of the permeation coefficient of the pure PVA *P*_PVA_ and that of the nanocomposite *P*_NC_.


(6) $$\begin{array}{l} BIF=\frac{P_{\text{PVA}}}{P_{\text{NC}}} \end{array}$$

#### 2.2.4. Measurement of Helium Permeability

The helium permeability was measured with a permeameter QHV-4 (Vinci Technologies, Nanterre, France) using a patented protocol (46, 47). In this measurement device, a mass spectrometer detects an ion current proportional to the permeating rate of the gas. The measurement is performed at 38°C and 0 % RH. First, a helium calibrated leak is measured, then the baseline, and finally, the helium transmission rate of the sample. The measurement is automatically stopped in the steady state considering a geometrical criterion. The use of helium gas allows reducing the measurement time, which is in the range of 1 h considering the full procedure (47).

#### 2.2.5. Comparison of Experimentally Measured Permeability Values of Filled Polymer Systems With Values Calculated Based on Theoretical Models

**Figure 1:**
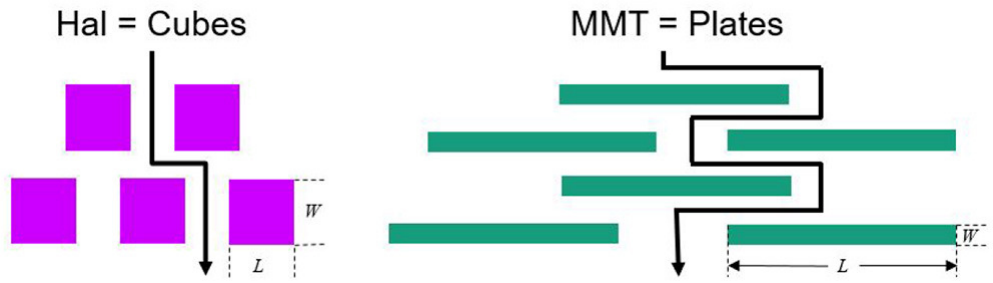
2D model for the path of a diffusing molecule through a polymer filled with either impermeable sticks (Hal) or impermeable platelets (MMT), redrawn from (48).


The Nielsen model was used in order to calculate the permeability coefficients and compare these with the experimentally measured values (48). It is a purely geometrical model assuming that the nanoparticles are impermeable for the permeating gas and, therefore, increase the diffusion path length, called tortuous path, as shown in Figure 1. It is assumed in this model that all particles are homogeneously dispersed and fully exfoliated with no agglomeration in the polymer layer and all oriented parallel to the surface, which means perpendicular to the effective direction of permeation. Although the permeation process through a filled polymer is an extremely complex phenomenon, the model allows—with some assumptions—the appraisal of the permeation blocking potential of nanoparticles based on the geometrical factors. These factors are the particle length *L*, the particle width *W*, and the volume ratio of the nanoparticle (filler) ϕ_F_. The tortuosity τ is defined as (49).

(7) $$\begin{array}{l} \tau =\frac{\delta }{d} \end{array}$$

where δ is the distance a molecule must travel to get through the film, i.e., the diffusion path length, and *d* is the unit length (thickness) of the film. According to this model, δ_theo_ is calculated *via* Equation (8), based on the particle size and volume ratio of the filler (48).


(8) $$\begin{array}{l} \delta_{\text{theo}}=\left(1+\frac{L}{2W}\cdot \phi_{\text{F}}\right)\cdot d. \end{array}$$

The empirical relation to evaluating δ is based on the measured values for the permeability of the filled (*P*_NC_) and the unfilled (*P*_PVA_) polymer (49).


(9) $$\begin{array}{l} \delta_{\text{emp}}=\sqrt{\frac{P_{\text{PVA}}}{P_{\text{NC}}}\cdot \left(1-\phi_{\text{F}}\right)\cdot d^{2}}. \end{array}$$

The two values δ_theo_ and δ_emp_, based on the particle geometry and the measured values for permeability, respectively, were compared. By knowing the values for *L*, *W*, and ϕ_F_, it is also possible to calculate the potential improvement for a known permeability value of PVA without filler according to Equation (10).


(10) $$\begin{array}{l} P_{\text{NC}}=\frac{1-\phi_{\text{F}}}{{\left(1+\frac{L}{2W}\cdot \phi_{\text{F}}\right)}^{2}}\cdot P_{\text{PVA}} \end{array}$$

#### 2.2.6. Scanning Electron Microscopy (SEM) Imaging of Barrier Films

The SEM images were captured with a JSM-7200F (JEOL, Akishima, Japan) at a high vacuum (2e-4 Pa). All samples were sputtered with a gold layer using a Hummer JR (Technics, Alexandria VA, USA), to reduce electrical charging of the non-conductive polymer samples. A solid state detector (SSD), detecting backscattered electrons, was used im combination with the JEOL-SEM software. The coated films were placed between Si wafers and cross-sections were prepared by Ar^+^ beam milling with a cross-section polisher (IB-19530CP, JEOL, Japan).

#### 2.2.7. X-ray Diffraction

The X-ray powder diffraction (XRD) data were collected with a Seifert 3003 TT diffractometer (XRD Eigenmann GmbH, Schnaittach-Hormersdorf, Germany) using Cu-K_α_ radiation (λ = 1.54184 Å) equipped with a METEOR 1D linear detector in Bragg-Brentano geometry.

#### 2.2.8. Fourier Transform Infrared (FTIR) Spectroscopy

The FTIR spectra were measured with a Frontier™ FTIR spectrometer L1280034 and analyzed with the PerkinElmer Spectrum IR, Version 10.6.1 software, both supplied by PerkinElmer (Shelton CT, USA). A golden gate setup was used to fix the coated films with the coated side to the diamante crystal for the ATR (attenuated total reflectance) measuring mode. A total number of 16 scans with a resolution of 4 $\frac{\text{1}}{\text{cm}}$ were recorded within a wavenumber range of 4.000 to 600 $\frac{\text{1}}{\text{cm}}$. All spectra were baseline corrected.

## 3. Results and Discussion

### 3.1. Particle Size Distribution and Aspect Ratio

Since the isolation and a simultaneous rotation of a single silicate platelet or a tube, in order to be able to measure the extension in three dimensions, is unfeasible, the size estimation of the nanoparticles was executed in two steps. To determine *L* and *T* (x- and y-direction), the exfoliated MMT in dispersion was analyzed using AF4/MALLS. To measure the extension in the z-direction (*W*), SEM images of cross-sections of the coatings were analyzed. The assumption for this is that the degree of exfoliation of the nanoparticles is not effected during the mixing process of the dispersion with PVA (20).

**Figure 2:**
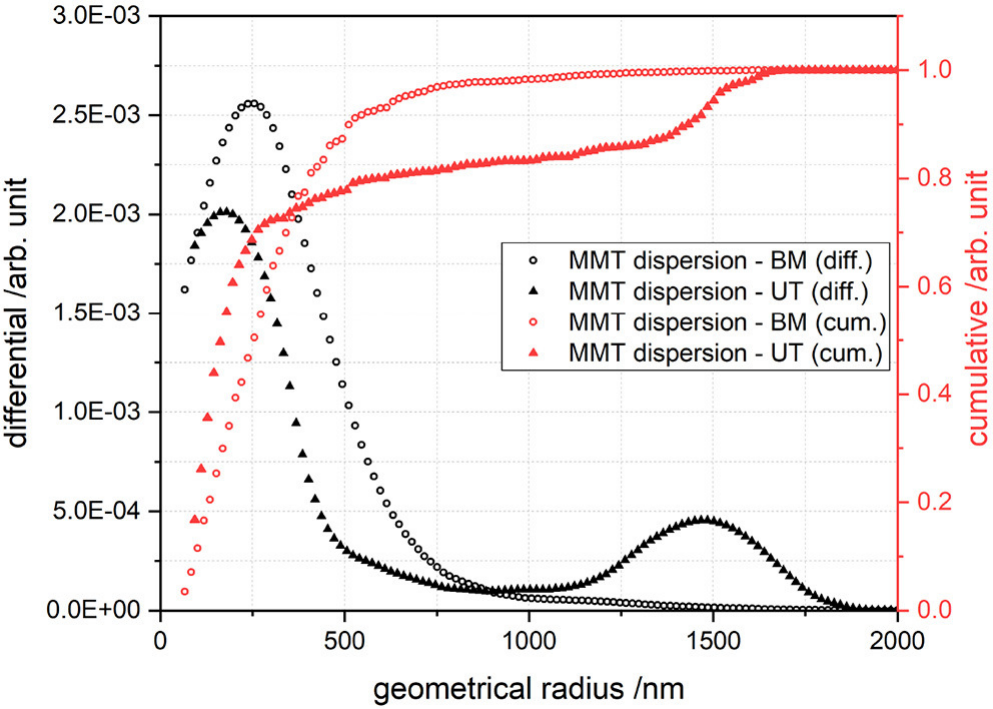
Representative measurement of particle size distribution, assuming spherical shaped particles, for MMT in aqueous dispersion mixed with ULTRA-TURRAX^®^ and planetary ball mill.

**Figure 3:**
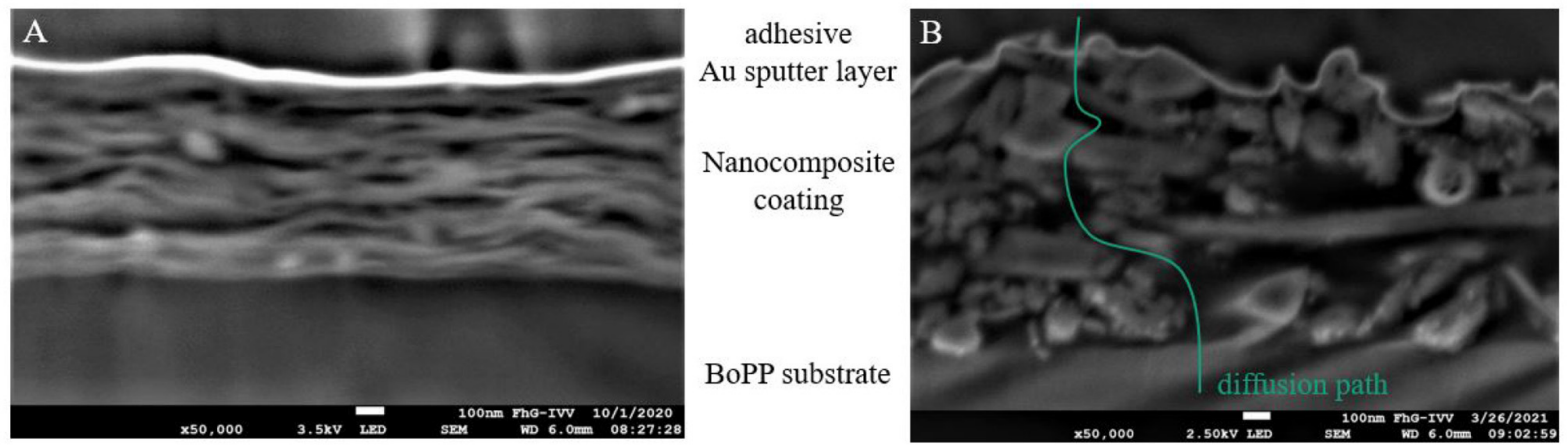
Scanning electron microscopy (SEM) images of cross-sections prepared by Ar polishing of nanocomposite coatings with MMT-BM-30 **(A)** and Hal-BM-30 **(B)** with a magnification of 50.000; from bottom to top the layers are the BoPP substrate, the nanocomposite coating, the sputtered gold layer, and the top layer is the adhesive used to bond it to the Si wafer; in the nanocomposite layers the white areas are the nanoparticles and the black areas are PVA; for the Hal-containing sample **(B)** diffusion paths, meaning an end-to-end line with no particles, perpendicular to the coating, can be found (one example shown in green), whereas for the MMT-containing sample the diffusion path in this 2D image is always blocked by MMT platelets.

Figure 2 shows the size distribution of silicate platelets dispersed in water as a function of *r*_geo_ calculated using Equation (1). The particle radius is then transferred to particle size *L* (=*T*) by Equation (2). For the dispersion with BM, 90 % of the particles are smaller than 1,000 nm, which is represented by the cumulative curve. The average value for particle size for this dispersion technique was around 494 nm. The dispersion with the UT, on the other hand, showed two peaks, 90 % of the particles were smaller than 3.000 nm with two appearing sizes at 340 and 2,960 nm. The particles bigger than 2.000 nm, originated from agglomerates of silicate platelets that were not exfoliated sufficiently. Nevertheless, the average value for *L* (=*T*) of the exfoliated particles was found to be 340 and 494 nm prepared by UT and BM, respectively. The particles were not broken while dispersing with BM, despite the countless crushes with the ZrO balls. Using the AF4 technique, it is possible to determine the particle size, if the particles appear as spheres, but since they are platelets, cross-section images were analyzed to determine their width. Figure 3A shows a cross-section image of MMT-BM coating. In this illustration the MMT platelets appear to be white, since the electron density of silicates is higher than it is for PVA due the heavier Al, Si, and Mg atoms. The thickness of these white lines indicates the *W* of the platelets and a value of 25 nm was measured for both MMT-BM and MMT-UT (not shown here) samples. Hence, the aspect ratio (*L*/*W*) of the MMT particles was calculated as 14 and 20 for dispersions prepared by UT- and BM-processes, respectively. These values are at the lower end of a range from 10 to 1,000, reported for the aspect ratio of MMT containing clay particles (50–54). The variations in *L*, *T*, and *W*, leading to this broad range, are due to the differences in natural abundance in clay minerals, as well as the different preparation methods. Whereas high barriers are normally reached with high aspect ratios (48, 49, 55), nanolacquers with these particles are limited in viscosity, total solid content, and mixing ratio (54). That means an increased size in the aspect ratio always goes along with an increase in viscosity of the nanolacquer. The viscosity, however, can only be as high as the coating technique allows. Additionally, a higher aspect ratio requires more water molecules to be part of the hydration shell, which is necessary for a stable dispersion. Therefore, less water molecules are available to dissolve the polymer, which results either in a lower total solid content of the lacquer or in a limitation in the mixing ratio (56). The parameters of aspect ratio, mixing ratio, total solid content, and viscosity, need to meet the requirement of the coating technique and intended number of coatings and coating layer thickness. The nanoparticles with aspect ratios of 14 and 20, used in this study, allow exactly this sufficient solid content and mixing ratio for the coating within one process step with the k-bar coating technique.

**Figure 4:**
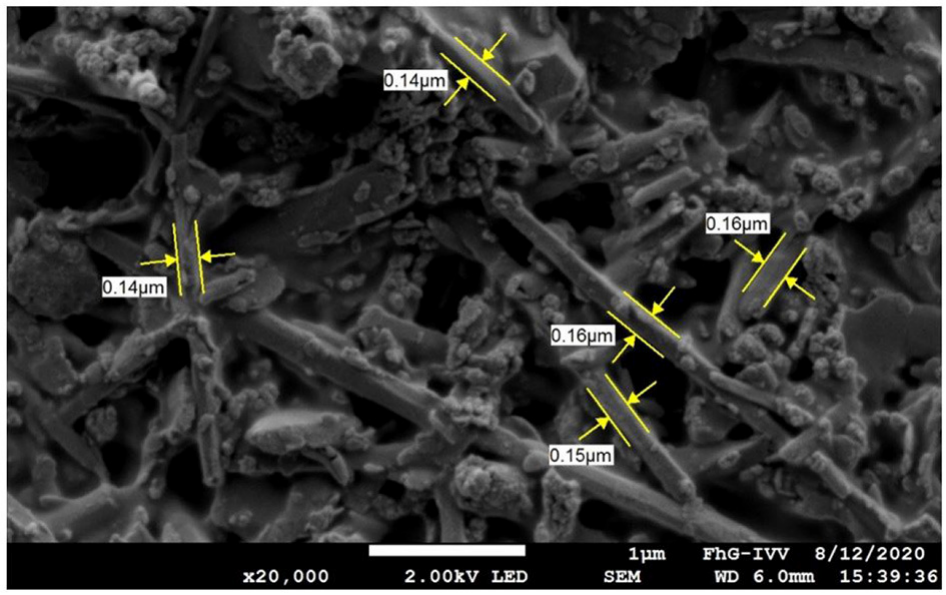
Top-down SEM image with a magnification of 20.000 of the Hal-BM-47 coating; the sticks are Hal particles, whose size *L* (=*W*) is measured; the lumps next to the sticks are PVA; at a filler ratio of 47 vol% there is no coherent structure anymore.

It was not possible to perform the size measurement for Hal with the AF4 technique, since the Hal particles were sedimenting too fast in an aqueous dispersion. Therefore, only SEM images were used to determine the *L* and *W* of the Hal sticks. As shown in the top view image in Figure 4, a value of 150 nm for both extensions of *L* and *W*, which represent the diameter in the stick-shaped geometry of Hal, was used for aspect ratio calculation. This accords with reported values for the outer diameter of Hal nanotubes (50 to 200 nm) (57, 58). The aspect ratio in 2D, as defined by Nielsen (48) is, therefore, found to be 1 for Hal particles.

### 3.2. Oxygen Permeability

**Figure 5:**
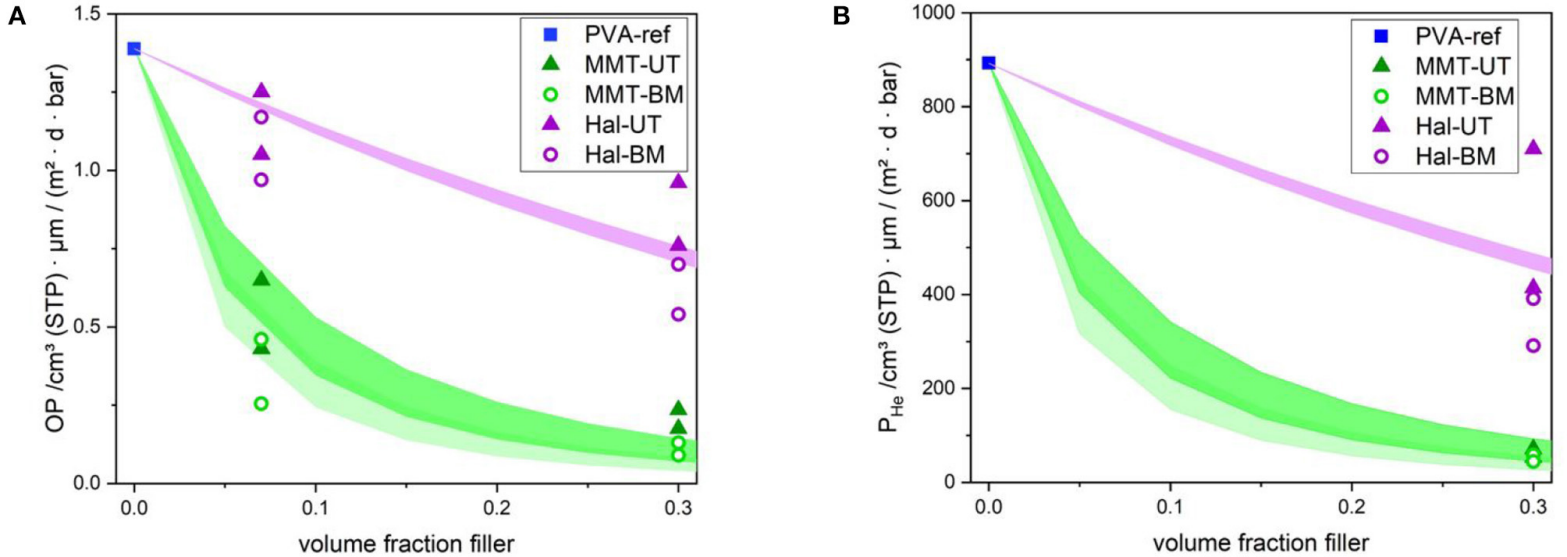
Calculated and measured oxygen **(A)** and helium **(B)** permeability values as a function of the nanoparticle concentration in volume. A 2-fold measurement for PVA, MMT-UT, MMT-BM, Hal-UT, and Hal-BM was executed, where both values for each sample are shown. The areas marked in color represent the calculated values for permeability coefficients of nanocomposites according to Equation (10). The particle size ranges used for MMT-UT were 320–360 nm for particle length and 20–30 nm for particle width (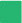 MMT-UT). The size ranges used for MMT-BM were 474–514 nm for particle length and 20–30 nm for particle width (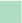 MMT-BM). The size ranges used for Hal were 140–160 nm for tube diameter (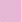 Hal, independent dispersion technique).

Figure 5A shows the experimentally determined values for the oxygen permeability coefficients, *P*_O_2__, only for the coating layers, calculated according to Equations (4) and (5). The transmission rate of the BoPP substrate film was found to be 1.250 $\frac{{\text{cm}}^{3}\;\left(\text{STP}\right)}{{\text{m}}^{2}\;\text{d}\;\text{bar}}$. The dry coating layer thicknesses were (1.0 ± 0.2) μm for the layers with 30 and 47 vol% and (0.6 ± 0.1) μm for the layers with 7 vol% of nanoparticles. The PVA-ref sample provided a dry coating layer thickness of (1.0 ± 0.2) μm.

The oxygen permeability coefficient of PVA depends on various parameters. Values of 1 to 6 $\frac{{\text{cm}}^{3}\;\left(\text{STP}\right)\;1\;\mu \text{m}}{{\text{m}}^{2}\text{d}\;\text{bar}}$ are reported in the literature for coatings comparable to those in this study (14–18). The measured value of 1.4 $\frac{{\text{cm}}^{3}\;\left(\text{STP}\right)\;1\;\mu \text{m}}{{\text{m}}^{2}\text{d}\;\text{bar}}$ in this study is within that range. Since PVA resin composition and type, the coating method, and the conditions used during coating and drying of pure PVA were kept similar to those used for the coatings with nanolacquer, the measured oxygen permeability coefficient for pure PVA, *P*_O_2_|*PVA*_, was used in Equation (6) to calculate the *BIF*_O_2__. The highest barrier improvement factor has been achieved with the medium volume concentration of 30 vol% silicate, followed by the lowest volume concentration of 7 vol%. At the highest volume concentration of 47 vol%, no barrier improvement was obtained. The addition of only 7 vol% platelet-shaped MMT particles already led to an improvement of *P*_O_2_|*NC*_ to 0.55 and 0.36 $\frac{{\text{cm}}^{3}\;\left(\text{STP}\right)\;1\;\mu \text{m}}{{\text{m}}^{2}\text{d}\;\text{bar}}$ for MMT dispersed *via* UT and BM, respectively. The *BIF*_O_2__ values are, therefore, 3.9 and 4.4. A further increase in the volume ratio of MMT to 30 vol% led to a further decrease in the permeability to 0.21 and 0.12 $\frac{{\text{cm}}^{3}\;\left(\text{STP}\right)\;1\;\mu \text{m}}{{\text{m}}^{2}\text{d}\;\text{bar}}$ for UT and BM dispersion, respectively. These are the lowest *P*_O_2_|*NC*_ values achieved in this study, which is also represented by the relatively high *BIF*_O_2__ values of 6.8 for UT and 12 for BM dispersed samples. However, further increasing the amount of MMT did not constantly lead to an improvement: for instance, at a concentration of 47 vol% MMT, a strong increase in *P*_O_2_|*NC*_ compared to pure PVA was reached. The *P*_O_2_|*NC*_ was measured as 76 $\frac{{\text{cm}}^{3}\;\left(\text{STP}\right)\;1\;\mu \text{m}}{{\text{m}}^{2}\text{d}\;\text{bar}}$ for MMT-UT-47 and 50 $\frac{{\text{cm}}^{3}\;\left(\text{STP}\right)\;1\;\mu \text{m}}{{\text{m}}^{2}\text{d}\;\text{bar}}$ for MMT-BM-47. Apparently, there is no coherent structure anymore with this high filler concentration. The values are not shown in the diagram.

Looking at the *BIF*_O_2__ values more closely, it is noticeable that they do not differ significantly between the UT and BM dispersion techniques at a volume fraction of 7 %. However, at a volume fraction of 30 %, the *BIF*_O_2__ is higher for BM than for UT dispersed samples (refer to Table 3). The higher the solid content of a dispersion, the more difficult it is to distribute the agglomerates completely, during dispersal. The BM process provides, in comparison to the UT process, a more homogeneous shearing, since the whole dispersion is equally in contact with the milling zirconium oxide balls (33), whereas the UT has a rotational mixing head, which is located in the middle of the dispersion during mixing (34). This study has found that at a high concentration of 30 vol%, this uniformity of distribution can be sustained better by BM dispersion technique (refer to Section 3.1). The present agglomerates after UT dispersion technique, on the one hand, disturb the parallel alignment of the exfoliated platelets sterically and, on the other hand, the agglomerated platelets do not contribute to the elongation of diffusion path. These reasons, together with the slightly smaller particle size after UT dispersion, cause the lower *BIF*_O_2__ for UT dispersed samples at 30 vol%.

**Table 3:** The aspect ratio of nanoparticles, gas barrier improvement factors of nanocomposites for helium and oxygen, and comparison of molecule travel distance, δ is given.

	**Aspect ratio**	**Helium**				**Oxygen**			
**Sample code**	***L*/*W***	***BIF*_He_**	**δ_theo_**	**δ_emp_**	**Difference**	***BIF*_O_2__**	**δ_theo_**	**δ_emp_**	**Difference**
			**μm**	**μm**	**%**		**μm**	**μm**	**%**
***n***	**5**	**2**	**5**	**2**		**2**	**5**	**2**	
MMT-UT-30	14 ± 2.7	14 ± 3.0	2.7 ± 0.8	2.9 ± 0.7	7.0	6.8 ± 2.3	2.7 ± 0.8	2.0 ± 0.6	26
MMT-BM-30	20 ± 4.0	17 ± 2.0	3.6 ± 0.8	3.1 ± 0.8	14	12 ± 3.0	3.6 ± 0.8	2.6 ± 0.7	28
Hal-UT-30	1.0 ± 0.1	1.6 ± 0.3	1.0 ± 0.1	0.9 ± 0.2	n.a.^*^	1.6 ± 0.3	1.0 ± 0.3	1.0 ± 0.3	n.a.^*^
Hal-BM-30	1.0 ± 0.1	2.9 ± 0.8	1.0 ± 0.1	1.3 ± 0.4	n.a.^*^	2.2 ± 0.8	1.0 ± 0.1	1.1 ± 0.5	n.a.^*^

*The values are given with SD for the number of measured specimens n per sample*.

* *No tortuous path effect for Hal*.

As expected, the *BIF*_O_2__ values for platelet-shaped particles were found to be remarkably higher than for stick-shaped ones. The addition of 7 vol% Hal led to a slight improvement, resulting in an oxygen permeability coefficient of 1.1 and 1.2 $\frac{{\text{cm}}^{3}\;\left(\text{STP}\right)\;1\;\mu \text{m}}{{\text{m}}^{2}\text{d}\;\text{bar}}$ for UT and BM, respectively, compared to 1.4 $\frac{{\text{cm}}^{3}\;\left(\text{STP}\right)\;1\;\mu \text{m}}{{\text{m}}^{2}\text{d}\;\text{bar}}$ for pure PVA. The lowest permeability values reached by the addition of Hal were achieved for a filler ratio of 30 vol% and were 0.87 and 0.63 $\frac{{\text{cm}}^{3}\;\left(\text{STP}\right)\;1\;\mu \text{m}}{{\text{m}}^{2}\text{d}\;\text{bar}}$ for UT and BM dispersion technique, respectively. Similar to the platelet-shaped particles, a further increase of the volume fraction above 30 vol% led to a reduction in barrier properties.

In general, the effects of the mixing ratio and the dispersion technique on the gas barrier enhancement for both particles types, MMT and Hal, were found to be similar. The highest barrier was achieved for a mixing ratio of 30 vol% and the BM dispersion technique led to the higher barrier performance, due to the reduced amount of agglomerations.

The reason for not having a high barrier improvement with Hal, refer to Table 3, was that there is not really a tortuous path created by the integration of sticks. As shown in the cross-section image of the coating, Figure 3B, the sticks only acted as small obstacles but did not really build up a closed layer with an elongation of the permeation path. The alignment and homogeneous distribution of the platelet-shaped particles, which is an assumption of the Nielsen model, is demonstrated in the cross-section SEM image in Figure 3A. The MMT particles, indicated by the white lines, are oriented parallel to the surface and provide a tortuous path. This SEM image indicates the tortuous path effect for the 30 vol% MMT nanocomposite prepared by the BM dispersion technique, as it was also shown schematically in Figure 1.

Based on this, further investigations were performed with the samples containing 30 vol% nanoparticles, due to their higher *BIF*_O_2__ values.

### 3.3. Helium Permeability

Roberts et al. (29) proposed a model for permeation through oxide layers (e.g. AlO_x_) coated via physical vapor deposition. Within this model, defects or pinholes in the oxide layer with a size of ≥1 nm are defined as macro-defects, and defects or pinholes with a size of 0.35 to 1 nm are defined as nano-defects. This model has been transferred to the permeation in nanocomposites. In this study that is about the investigation of nanocomposites the expression “defect” does not describe a pinhole in a very thin oxide layer, but an inhomogeneity in the coated layer, e.g. a not parallel aligned particle or an agglomeration that disturbs the homogeneous distribution of the particles. The defect does not necessarily be an air-filled hole, as it can also be described as a free volume with no inorganic particle, but with polymer.

It is known that small molecules, such as water, permeate predominantly through nano-sized defects (30, 31). However, water molecules form hydrogen bonds with the side groups of PVA and disrupt the intermolecular hydrogen bonds among the PVA chains (59, 60). Hence, helium, being an inert gas, was chosen as the small permeating molecule. On the other hand, it is known that for oxygen, the permeation mechanism is dominated by macro-sized defects, i.e., regions where there is no inorganic material (29, 31). Therefore, helium (2.6 Å) and oxygen (3.5 Å), with two different gas kinetic diameters, were compared in terms of their permeability in the nanocomposites, in order to distinguish the dominating structural effects on the gas permeation (28).

The measured permeability coefficients for helium, *P*_He_, are shown in Figure 5B. The *P*_He|PVA_ for pure PVA was 892 $\frac{{\text{cm}}^{3}\;\left(\text{STP}\right)\;1\;\mu \text{m}}{{\text{m}}^{2}\text{d}\;\text{bar}}$, which is close to the value of 765 $\frac{{\text{cm}}^{3}\;\left(\text{STP}\right)\;1\;\mu \text{m}}{{\text{m}}^{2}\text{d}\;\text{bar}}$ measured by Carstens and Ehart (61). The integration of 30 vol% MMT particles reduces the *P*_He_ down to 62 and 51 $\frac{{\text{cm}}^{3}\;\left(\text{STP}\right)\;1\;\mu \text{m}}{{\text{m}}^{2}\text{d}\;\text{bar}}$ for UT and BM dispersion techniques, respectively. There is no significant difference between the barrier performance of the samples prepared by UT and BM dispersion techniques, where the *BIF*_He_ was calculated as 14 and 17, respectively. An integration of stick-shaped Hal particles, however, only led to a slight helium barrier improvement. The *BIF*_He_ is found to be 1.6 and 2.9 with the corresponding *P*_He|NC_ values of 565 and 341 $\frac{{\text{cm}}^{3}\;\left(\text{STP}\right)\;1\;\mu \text{m}}{{\text{m}}^{2}\text{d}\;\text{bar}}$ for UT and BM dispersion, respectively.

**Figure 6:**
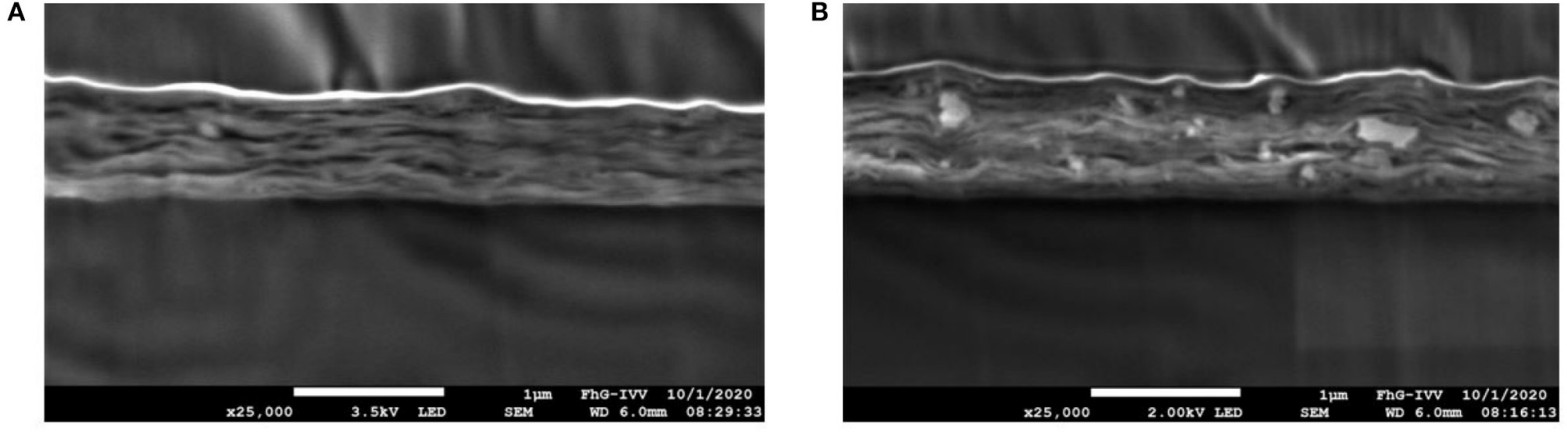
Scanning electron microscopy images of cross-sections with a magnification of 25.000 prepared by Ar polishing of MMT-containing samples dispersed with a planetary ball mill **(A)** and ULTRA-TURRAX^®^ **(B)**; from bottom to top the layers are the BoPP substrate, the nanocomposite, the sputtered gold layer, and the adhesive to bond the Si wafer; for the BM-dispersed sample **(A)**, there are nearly no agglomerations in the layer, whereas for the UT-dispersed samples **(B)**, some agglomerations are present, which lead to disturbances in the homogeneity of the layer.

Unlike the oxygen permeation, for the helium permeation, there was no significant difference between the *BIF*_He_ values of MMT-UT-30 and MMT-BM-30 formulations (refer to also Table 3). This might be due to the fact that an agglomeration essentially affects the macro-structure of a coating layer but only slightly affects the nano-structure. Wang et al. (62) showed that agglomerations of MMT particles lead to a reduced oxygen barrier performance because of poor inter-facial compatibility. That means an agglomeration disturbs the homogeneous distribution and alignment of the platelets in the layer. This disturbance might lead to additional free volumes or areas with no inorganic particle and adversely oriented particles (63, 64). The macro-sized defects enable the oxygen to permeate, why the UT-dispersed sample only provided a *BIF*_O_2__ of 6.8 for oxygen. The BM-dispersed sample, however, provided a *BIF*_O_2__ of 12, since there were fewer disturbances of the particle alignment due to fewer agglomerations as a result of the high shear ball milling process as shown in Figure 6 and by the size measurement results in Section 3.1.

Similar to the oxygen permeation, the Hal-containing nanocomposites did not provide a barrier improvement for helium.

### 3.4. Crystallinity and Intermolecular Interactions

Unlike the permeation through an oxide layer or the coating with simple barrier lacquer, a nanocomposite is a two-material system, meaning the components can interact and affect each other. To evaluate this interaction between PVA and nanoparticles, XRD and FTIR measurements have been performed, and the activation energy of the permeation coefficient was determined.

**Figure 7:**
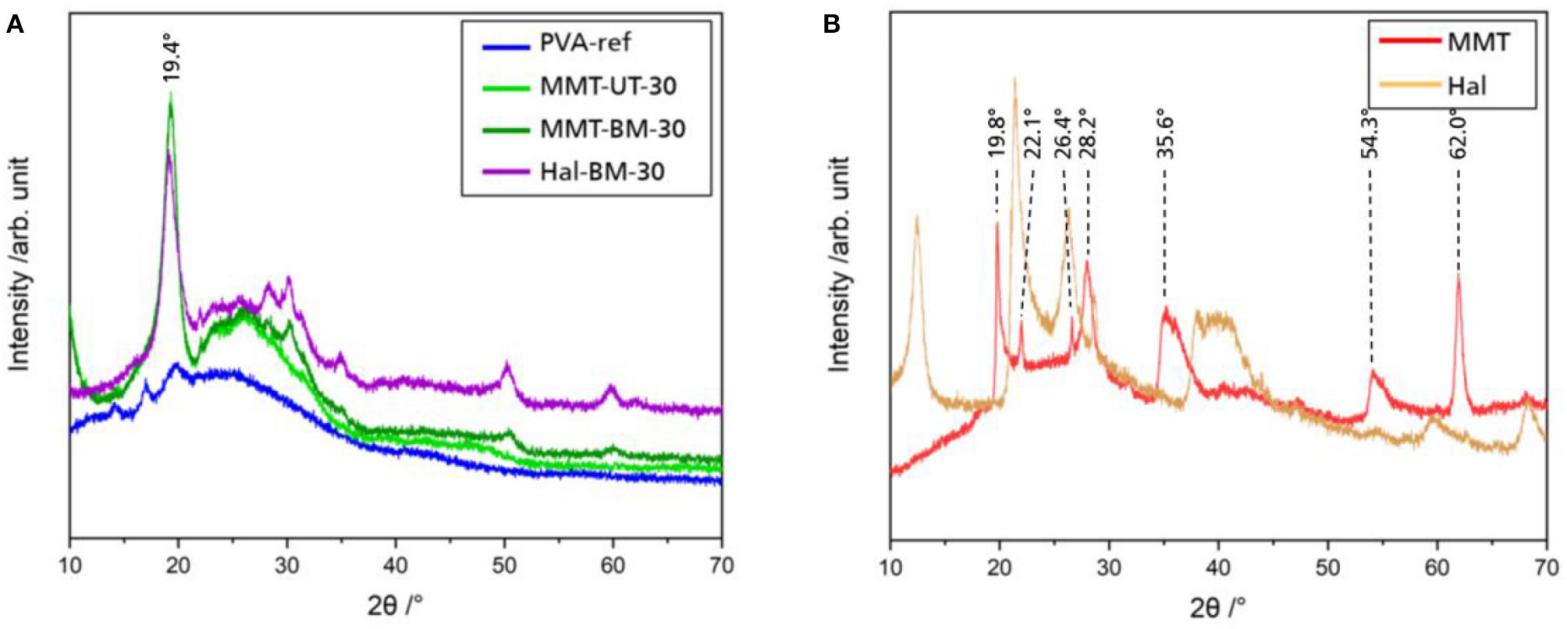
X-ray diffraction (XRD) pattern of free-standing films of PVA, MMT, and Hal nanocomposites **(A)** and of pure MMT and Hal **(B)** as a function of a 2Θ range of 10 to 70°.

X-ray powder diffraction measurements were performed for pure MMT, pure Hal, free-standing PVA film, and free-standing nanocomposite films to evaluate the effect of clay particles on the crystallinity of PVA. The diffraction pattern is shown in Figure 7. The pure PVA appeared with the typical peak shape of a semicrystalline material, which stresses a high amount of amorphous ratio with only a small reflection at 19.4° and a broad maximum called a hump. The nanocomposite also appeared with the peak shape of a semicrystalline material, but with a larger quantity of crystalline phase indicated by the larger reflection at 19.4° (65). The reflection at 19.4°, which corresponds to the $10\bar{1}$ reflection, is present for both, pure PVA and nanocomposite, and is typical for crystalline atactic PVA (66, 67). The diffraction pattern of pure MMT provides a reflection at 19.8°, which is quite close to this atactic reflection. However, the other reflections of MMT, which were in good accordance with previously reported ones (68), at 22.1, 26.4, 28.2, 35.6, 54.3, and 62.0°, were not observed in the nanocomposite curve, which indicates that PVA is the dominating part in the nanocomposite's diffraction pattern. The same is valid for the Hal-containing sample (69). To estimate the degree of crystallinity of one sample, the ratio between the amorphous hump and crystalline peak within one curve is examined. For the nanocomposites, the percentage of the crystalline peak is higher than for the PVA film. Hence, the addition of clay particles to the PVA solution seemed to increase the crystallinity of the PVA matrix.

In the literature, there is no clear trend discernible when it comes to the effect of nanoparticle integration on polymer crystallinity. There are some publications claiming that the crystallinity decreases (12, 70), whereas other authors report that the crystalline fraction increases (71–73). The decrease in crystallinity is explained by a disturbance of crystalline regions in PVA by clay particles. On the contrary, the clay particles may act as crystallization nuclei, which would lead to an increase in the number of crystalline regions, and therefore, the total crystallinity. Zhu et al. (74) showed that the influence on the crystalline appearance of nanocomposites is highly dependent on the curing conditions and, therefore, the crystallization kinetics. Consequently, the measurements in this study were performed with the pure PVA and nanolacquer using the same coating and curing conditions for both, to see the influence of clay particles on crystallinity and intermolecular interactions. An increase in matrix crystallinity leads to an improvement of the gas barrier of the polymer (4, 17). Since the crystallinity of Hal-containing samples has increased in the same order of magnitude, as the MMT-containing samples (refer to Figure 7), the higher *BIF*_He|O_2__ is mainly based on the geometrical factor, i.e., the tortuous path effect. The very slight increase of the barrier properties of Hal-containing samples, however, might be based on the increase in crystallinity.

**Figure 8:**
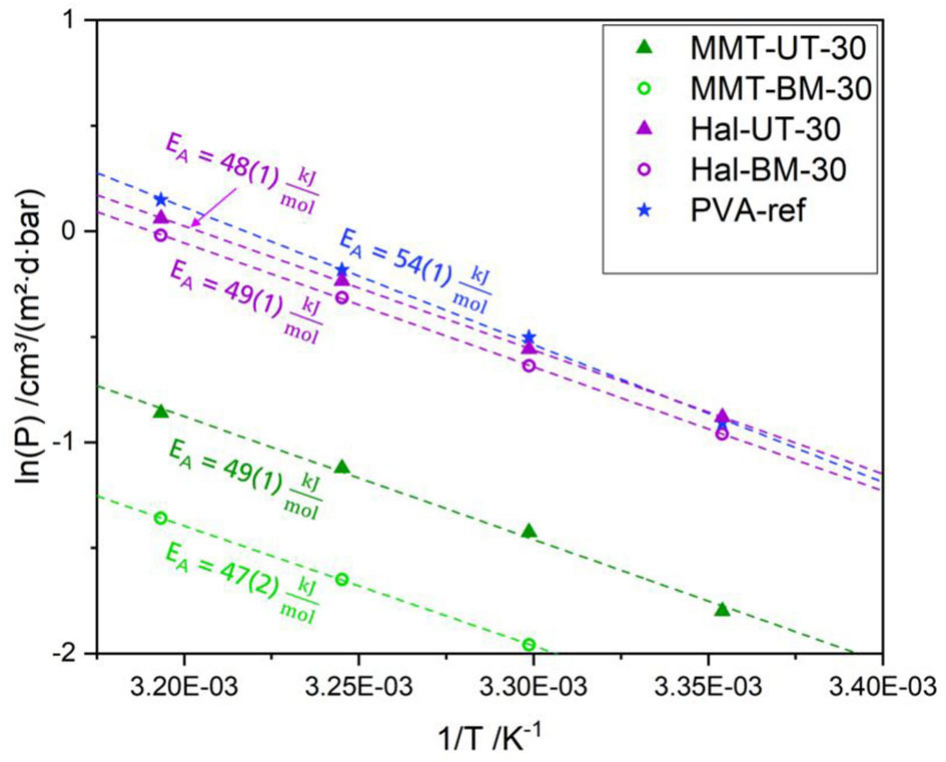
Arrhenius plots for determining activation energy of oxygen permeation of PVA-ref, MMT-UT-30, MMT-BM-30, Hal-UT-30, and Hal-BM-30. The given values for the activation energy are calculated according to Equation (3) with SD for a 2-fold measurement.

The activation energy for the permeation coefficient measured for pure PVA was found to be higher than the values measured for all nanocomposites, and there was no significant difference among themselves as shown in Figure 8.

The highest activation energy with 54 $\frac{\text{kJ}}{\text{mol}}$ was measured for pure PVA coatings, which fits quite well with the value measured by Zhang et al. (75). With the integration of 30 vol% silicate nanoparticles, the activation energy decreased down to 47 $\frac{\text{kJ}}{\text{mol}}$ for the nanocomposites, being very similar to each other regardless of the mixing method and the particle shape. This reduction in the activation energy values of the nanocomposite in comparison to pure PVA was expected to be due to the weakening of the intermolecular hydrogen bonds of the PVA chains. As proven by the following FTIR investigation, the silicate particles interact with the PVA matrix, probably *via* hydrogen bonds. If some hydroxy groups of a PVA chain are interacting with an adjacent silicate particle, these hydroxy groups are not part of the intermolecular cohesion of the PVA matrix anymore. This share of hydroxy groups in the adjacency of particles and PVA chains leads to weak boundary areas where the diffusion might take place more easily, as for example proposed by Bhunia et al. (76).

**Figure 9:**
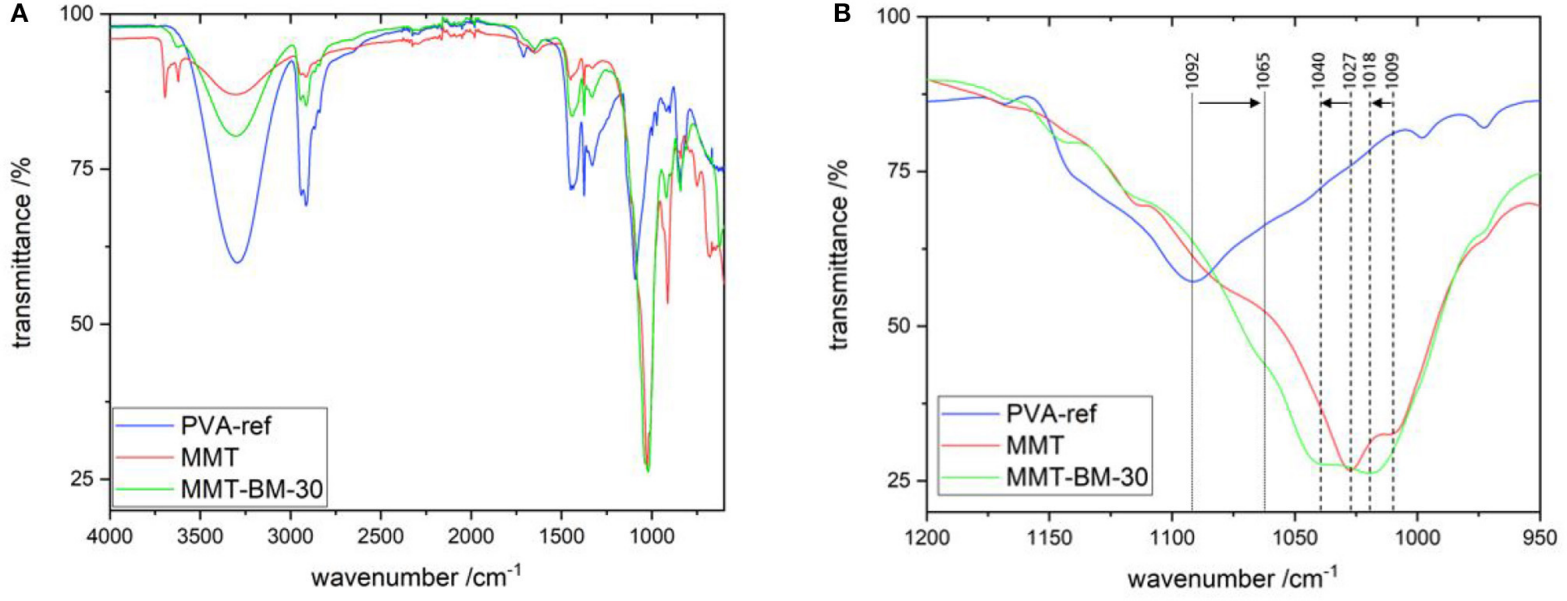
Fourier transform infrared (FTIR) spectra, **(A)** measured in the range from 500 to 4000 1/cm for PVA, pure MMT, and MMT-BM-30. **(B)** Shows a magnification of the fingerprint region and indicates the shift of the C-O (1092 to 1065 1/cm) and the Si-O (1027 to 1040 1/cm and 1009 to 1018 1/cm) stretching mode of PVA and MMT, respectively. MMT-UT-30 provides similar curve as of MMT-BM-30 and is not shown in the figure.

The intermolecular interaction between PVA and MMT nanoparticles was determined by means of FTIR measurements (see Figure 9). This interaction involves hydrogen bonds between the hydroxy groups of PVA and the terminal oxygen atoms of the Si-tetrahedra. In MMT-BM-30, this was indicated by a shift of the C-O stretching mode of PVA from 1092 $\frac{\text{1}}{\text{cm}}$ to 1065 $\frac{\text{1}}{\text{cm}}$ and a shift of the Si-O stretching mode of MMT from 1027 $\frac{\text{1}}{\text{cm}}$ to 1040 $\frac{\text{1}}{\text{cm}}$ and 1009 $\frac{\text{1}}{\text{cm}}$ to 1018 $\frac{\text{1}}{\text{cm}}$, respectively (77, 78). Since the positions of the C-H stretching vibration (2941 and 2808 $\frac{\text{1}}{\text{cm}}$) and the C-H deformation (1375 $\frac{\text{1}}{\text{cm}}$) peaks, originating from the PP substrate film, retained their positions for all three measurements, a peak shift of mentioned peaks from PVA and MMT was present and not a shift of the whole curve (79, 80). The combined peak of C-O and Si-O stretching modes with signals at 1065, 1040, and 1018 $\frac{\text{1}}{\text{cm}}$ indicated this interaction between PVA and MMT, as well as the broadband in the region from 3600 to 3000 $\frac{\text{1}}{\text{cm}}$, which corresponds to the O-H groups involved in hydrogen bonds (71). It might be expected that the Si interactions with the PVA hydroxy groups reduce the inter-chain hydrogen bonds of PVA, which may, in turn, lead to cavity sizes within the PVA matrix being affected, thereby resulting in additional permeation paths for gas molecules.

In summary, the mixing of the MMT nanoparticles in a PVA polymer changed the crystallinity of the polymer and, on the other hand, caused the hydrogen bond to weaken, both seeming to have an opposing effect on the final gas permeability of the PVA matrix. Although no difference in these parameters was found for Hal and MMT particles, the MMT-containing samples showed a significantly higher BIF for both gases, suggesting that the tortuous path effect is the dominating factor for diffusion.

### 3.5. Comparison of Measured and Calculated Values for Gas Permeability and Diffusion Path Length

The permeation model for filled polymer systems developed by Nielsen is mainly based on particle geometry and particle concentration (48). That means, if the particle length *L*, the particle width *W*, and the amount of particles ϕ_F_ in a coating layer with thickness *d* are known, the permeability coefficient of a nanocomposite coating, *P*_NC_, can be calculated in comparison to its pure polymer matrix. In this study, this pure polymer matrix is PVA, having the permeability coefficient *P*_PVA_. Factors that are not considered in Nielsen's model are structural changes in the polymeric matrix of the nanocomposite induced by the nanoparticles possibly affecting its final gas permeability. The interaction of the PVA with silicate nanoparticles, leading to changes in PVA crystallinity and weakening of the intermolecular hydrogen bonds of the PVA, could have an effect on the gas permeability of the polymer within the nanocomposite. As suggested by Haghighi et al. (81), this limits the cohesion forces within the PVA and consequently decreases the degree of crosslinking. The possible effects of nanoparticles on these factors are discussed in Section 3.4.

The gas-blocking potential of the nanoparticles, calculated by Equation (10), is shown by the colored areas in Figure 5. The calculations are performed using the minimum and the maximum aspect ratio values for each concentration and are plotted; accordingly, the colored areas represent the regions in-between. The parameters used for the calculation of permeability coefficients for the respective dispersion technique, UT or BM, and the particle shape, Hal or BM, are listed in Table 4. The green and violet areas indicate the possible barrier improvement starting from pure PVA with 0 % filler up to a volume fraction of 30 %. The shape of these areas is found to be similar for both gases, helium and oxygen. For both particle shapes, platelets and sticks, the calculation provides an area due to the deviations in the parameters used; *L* and *W*.

**Table 4:** Parameters used for calculating *P*_NC_, δ_theo_, and δ_emp_ for oxygen and helium according to the Nielsen model Equations (8) and (9).

	**Length**	**Width**	**Filler ratio**	**Coating thickness**	**Permeability coefficients of pure PVA**	
	*L*	*W*	ϕ_F_	*d*	*P*_O_2|*PVA*__	*P*_He|PVA_
	nm	nm		μ m	$\frac{{\text{cm}}^{3}\;\left(\text{STP}\right)\;1\;\mu \text{m}}{{\text{m}}^{2}\text{d}\;\text{bar}}$	$\frac{{\text{cm}}^{3}\;\left(\text{STP}\right)\;1\;\mu \text{m}}{{\text{m}}^{2}\text{d}\;\text{bar}}$
*n*	5	5		5	2	2
MMT-UT-30	340^*^	25 ± 5	0 to 0.5	1.0 ± 0.2	1.4 ± 0.2	892 ± 47
MMT-BM-30	494 ^*^	25 ± 5				
Hal-UT-30	150 ± 10	150 ± 10				
Hal-BM-30	150 ± 10	150 ± 10				

*The values are given with SD for the number of measured specimens n per sample*.

* *Representative measurement with AF4/MALLS, refer to Figure 2*.

The calculated values fit quite well with the experimentally measured values for permeability coefficients. This is valid for both dispersion techniques and both particle shapes, indicating that the permeation process is dominated by the factors *L*, *W*, and ϕ_F_. Only in the case of oxygen permeation in the nanocomposite, with a volume filler fraction of 30 %, the difference between the experimental and calculated values is slightly higher. This can be due to the matrix changes, as discussed in more detail when comparing δ values.

Table 3 shows the values calculated for δ_theo_ and δ_emp_ using Equations (8) and (9), respectively, for 30 vol% samples. The values for δ_theo_ and δ_emp_ are compared using the measured dry layer thickness of the coating, which is (1.0 ± 0.2) μm for all the 30 vol% samples for both helium and oxygen. For a coating without nanoparticles, this dry layer thickness would be equal to the distance a molecule must travel. Both δ values for Hal-containing nanocomposites are found to be at 1 μm, similar to the dry coating layer thickness, verifying that stick-shaped particles do not provide a tortuous path effect in this configuration. The slight, but not outstanding, *BIF*_He_ (1.6 to 2.9) and *BIF*_O_2__ (1.6 to 2.2) of the two samples, Hal-UT-30 and Hal-BM-30, were not mainly the result of the tortuous path effect but were possibly due to the slight structural changes of PVA with nanoparticles, and Hal particles acting as obstacles within the PVA.

The calculated δ values for MMT-containing nanocomposites are found to be higher than for Hal-containing ones. The δ_emp|He_ values of 2.9 and 3.1 μm for UT and BM dispersed samples, respectively, fit well with the values for δ_theo|He_. This indicates that the distance a helium molecule must travel is elongated by a factor of around 3 when 30 vol% MMT are included in the nanocomposite. Similar behavior has also been observed for oxygen permeation. However, the values for δ_emp|O_2__ are, being 2.0 and 2.6 μm for UT and BM dispersed samples, respectively, found to be slightly smaller than for helium permeation. In this study, the elongation of the travel distance for oxygen is by a factor of 2 for UT and a factor of 2.6 for BM nanocomposites.

It might be that the nanoparticles lead to a change in the permeability coefficient of the PVA matrix within the nanocomposite, *P*_PVA_, due to an increase in the crystallinity, or weakening of the hydrogen bonds within the polymer matrix, or the interaction of the Si with the hydroxy groups of PVA (refer to Section 3.4). Since the difference between δ_theo|O_2__ and δ_emp|O_2__ is higher for oxygen (refer to Table 3), this change in *P*_PVA_ seems to affect mainly the bigger oxygen molecule and indicates additional oxygen permeation pathways within the nanocomposite.

## 4. Conclusions

This manuscript adds additional knowledge to the field of permeation mechanisms in nanocomposites. The main investigated factors are the effect of particle shape (sticks and platelets) on the tortuous path, the effect of the size of the permeating molecule (helium and oxygen), the effect of the polymer crystallinity on the permeability, and the comparison of calculated and measured permeability coefficients.

The permeability coefficients have demonstrated a barrier improvement factor of 17 for helium and 12 for oxygen, from the integration of 30 vol% platelet-shaped silicate particles dispersed with BM. For this particular nanocomposite, this corresponds to an elongation of the molecule travel distance by a factor of 3.1 for helium and a factor of 2.6 for oxygen. The preparation method for nanoparticles with a planetary ball mill has outperformed the method with ULTRA-TURRAX^®^, especially for oxygen.

There was no relevant barrier improvement measured for the nanocomposites with stick-shaped nanoparticles, neither for helium nor for oxygen. The molecule travel distance was not increased in comparison to the layer thickness of the nanocomposite because of their low aspect ratio.

The conformity of theoretical permeation values with the empirical ones affirmed the interpretation of the particle shapes' results. The permeation of the chosen gas molecules is affected by the geometrical factors, mainly the tortuous path effect.

The Nielsen model does not consider the structural effects of the nanoparticles on the PVA matrix. On the other hand, the XRD values showed an increase in the crystallinity of PVA due to the incorporation of nanoparticles. However, the FTIR investigation and the determination of the activation energy for permeation have shown an interaction between the silicate nanoparticles and the PVA polymer chains, which might lead to weaker boundary areas, with additional permeation pathways. These two contrary trends seem to oppose each other, which leads to good conformity of δ_theo_ and δ_emp_, especially for helium molecules.

Since the average value of δ_emp_ tends to be slightly lower than the value of δ_theo_, mainly for oxygen at 30 vol% MMT, and the weak boundary areas are present and not advantageous for permeation, there is still potential to improve the barrier performance, e.g., by an improvement of the chemical interaction of particles and polymer matrix by functional grafting of the particles. Another potential for investigation is the optimization of the aspect ratio. A one time coating step with a certain amount of solid content and a sufficient mixing ratio of polymer and particle should still be possible but with the highest aspect ratio of a commercially available particle. The integration of thin nanocomposite coatings, at layer thicknesses of 1 μm, will bring new insights for the production of recyclable flexible food packaging materials.

## Data Availability Statement

The raw data supporting the conclusions of this article will be made available by the authors, without undue reservation.

## Author Contributions

SS, EK, and PE contributed to conception and design of the study. OM and H-CL contributed especially to the permeation models and the described comparison of calculated and measured permeation values. SC performed the helium permeation measurement and contributed to the permeation discussion of oxygen and helium. All authors contributed to manuscript revision, read, and approved the submitted version.

## Funding

This study has received support from the BarriFlex project (IGF Nr. 235 EBG), funded by the Federal Ministry of Economic Affairs and Energy (BMWi) within the funding body Industrielle Gemeinschaftsforschung (IGF) *via* Arbeitsgemeinschaft industrieller Forschungsvereinigungen e.V. (AiF).

## Conflict of Interest

The authors declare that the research was conducted in the absence of any commercial or financial relationships that could be construed as a potential conflict of interest.

## Publisher's Note

All claims expressed in this article are solely those of the authors and do not necessarily represent those of their affiliated organizations, or those of the publisher, the editors and the reviewers. Any product that may be evaluated in this article, or claim that may be made by its manufacturer, is not guaranteed or endorsed by the publisher.
